# Neuroplastic effects of transcranial alternating current stimulation (tACS): from mechanisms to clinical trials

**DOI:** 10.3389/fnhum.2025.1548478

**Published:** 2025-03-12

**Authors:** Desmond Agboada, Zhihe Zhao, Miles Wischnewski

**Affiliations:** ^1^Department of Psychology, University of the Bundeswehr Munich, Neubiberg, Germany; ^2^Department of Biomedical Engineering, University of Minnesota, Twin Cities, MN, United States; ^3^Department of Psychology, University of Groningen, Groningen, Netherlands

**Keywords:** clinical trials, repeated stimulation, tACS (transcranial alternating current stimulation), neuroplasticity, long-term effects

## Abstract

Transcranial alternating current stimulation (tACS) is a promising non-invasive neuromodulation technique with the potential for inducing neuroplasticity and enhancing cognitive and clinical outcomes. A unique feature of tACS, compared to other stimulation modalities, is that it modulates brain activity by entraining neural activity and oscillations to an externally applied alternating current. While many studies have focused on online effects during stimulation, growing evidence suggests that tACS can induce sustained after-effects, which emphasizes the potential to induce long-term neurophysiological changes, essential for therapeutic applications. In the first part of this review, we discuss how tACS after-effects could be mediated by four non-mutually exclusive mechanisms. First, spike-timing-dependent plasticity (STDP), where the timing of pre- and postsynaptic spikes strengthens or weakens synaptic connections. Second, spike-phase coupling and oscillation phase as mediators of plasticity. Third, homeostatic plasticity, emphasizing the importance of neural activity to operate within dynamic physiological ranges. Fourth, state-dependent plasticity, which highlights the importance of the current brain state in modulatory effects of tACS. In the second part of this review, we discuss tACS applications in clinical trials targeting neurological and psychiatric disorders, including major depressive disorder, schizophrenia, Parkinson’s disease, and Alzheimer’s disease. Evidence suggests that repeated tACS sessions, optimized for individual oscillatory frequencies and combined with behavioral interventions, may result in lasting effects and enhance therapeutic outcomes. However, critical challenges remain, including the need for personalized dosing, improved current modeling, and systematic investigation of long-term effects. In conclusion, this review highlights the mechanisms and translational potential of tACS, emphasizing the importance of bridging basic neuroscience and clinical research to optimize its use as a therapeutic tool.

## 1 Introduction

Over the past century, several neuromodulation methods have been developed, with varying degrees of success. While such tools are used to test brain function in fundamental neuroscience research, sooner or later their potential therapeutic benefits must be explored in clinical studies. A clinically proven non-invasive neuromodulation technique for the treatment of major depressive disorder, obsessive-compulsive disorder, and more is transcranial magnetic stimulation (TMS) (Blumberger et al., 2018; Cole et al., 2020; Cohen et al., 2022). By providing repetitive magnetic impulses to a particular brain region, neuroplasticity can be induced, which may alleviate symptoms (Rossini et al., 2015; Lefaucheur et al., 2020). TMS is associated with minor side effects and is used in both advanced and mild stages of neurological and psychiatric disorders (Rossi et al., 2021). Treatments often last for 6–8 weeks, although more intense abbreviated therapies can finish within 1–3 weeks (Burke et al., 2019; Lefaucheur et al., 2020). A drawback of TMS is its significant costs, as well as the need for patients to go to a clinic for every intervention. Non-invasive low intensity electrical stimulation offers cheaper portable options that lend themselves well for home-based treatments. This includes transcranial direct current stimulation (tDCS), in which a subthreshold constant current is applied between two or more electrodes, with the goal of increasing or decreasing cortical excitability (Nitsche and Paulus, 2000; Jamil and Nitsche, 2017; Agboada et al., 2019). It is hypothesized that slight de- or hyper-polarization of resting membrane potentials are induced by anodal and cathodal tDCS, respectively (Stagg and Nitsche, 2011; Jamil and Nitsche, 2017). Consequently, the chance of naturally occurring neural firing is elevated or lowered. Another method, transcranial alternating current stimulation (tACS) applies an oscillating subthreshold current between two or more electrodes (Wischnewski et al., 2023). As tDCS, tACS affects the resting membrane potential, but rather than a consistent increase or decrease, it induces a fluctuation in the membrane potential, which can result in an elevated rhythmic neural firing (Krause et al., 2022; Wischnewski et al., 2024c). However, for these interventions to be successful clinically, their effects must outlast the stimulation duration. In other words, after-effects are crucial for the therapeutic efficacy of low intensity electrical stimulation. Various studies have demonstrated the physiological after-effects and clinical benefits of tDCS (Nitsche et al., 2008; Brunoni et al., 2012; Agboada et al., 2020; Mosayebi-Samani et al., 2023). These effects have been covered extensively elsewhere (Stagg and Nitsche, 2011; Jamil and Nitsche, 2017) and will thus not be discussed here.

The primary hypothesized effect of tACS is that neural firing and oscillations become entrained (Schutter and Wischnewski, 2016; Liu et al., 2018; Krause et al., 2023; Wischnewski et al., 2023). The external oscillatory current induces fluctuations in resting membrane potentials, which influences the rhythmic expression of naturally occurring neural spikes. These physiological effects have been demonstrated in a variety of studies (Fröhlich and McCormick, 2010; Ozen et al., 2010; Anastassiou et al., 2011; Krause et al., 2019; Johnson et al., 2020; Vieira et al., 2020; Huang et al., 2021; Krause et al., 2022; Vieira et al., 2024; Wischnewski et al., 2024c). However, in principle, this effect is restricted to the stimulation. When stimulation is turned off, the effect on the resting membrane potential and, thus, entrainment of neural firing evaporates. Nevertheless, a growing amount of studies have shown that tACS does have physiological, behavioral, and therapeutic after-effects (Kasten et al., 2016; Wischnewski et al., 2019a). In this review, we describe the potential mechanisms by which tACS may induce lasting effects. Further, we will discuss clinical studies using multisession tACS to inspect the longevity of lasting tACS effects.

## 2 Primary (online) neurophysiological effects of tACS

The alternating current applied to the scalp results in subthreshold oscillatory fluctuations in neural resting membrane potentials (Wischnewski et al., 2023). Consequently, neural firing becomes rhythmically locked to the external oscillation (Fröhlich and McCormick, 2010; Ozen et al., 2010; Anastassiou et al., 2011; Krause et al., 2019; Johnson et al., 2020; Vieira et al., 2020; Huang et al., 2021; Krause et al., 2022; Vieira et al., 2024; Wischnewski et al., 2024c). Meta-analytic evidence suggests that an e-field strength of 0.3 mV/mm is required for these effects to occur in at least a subset of neurons (Alekseichuk et al., 2022; Zhao et al., 2024). Furthermore, if neural firing is naturally entrained to a particular phase of a local field potential, tACS may result in a phase shift of the spiking (Krause et al., 2022; Vieira et al., 2024; Wischnewski et al., 2024c). To an extent, the online effects of tACS at the neural level translate to the macroscopic scale. For instance, motor cortical tACS has been shown to increase TMS-evoked muscle responses (Feurra et al., 2011, 2013; Cancelli et al., 2015; Heise et al., 2016; Cottone et al., 2018; Wischnewski et al., 2019a). Furthermore, studies that tracked the tACS phase online found that TMS-related motor-evoked potential (MEP) amplitudes were larger at particular phases (Guerra et al., 2016; Nakazono et al., 2016; Raco et al., 2017; Schilberg et al., 2018; Wischnewski et al., 2024c). While increases in oscillatory power during tACS stimulation have been suggested (Helfrich et al., 2014), it is important to consider that removing the tACS artifact from online EEG is difficult and there is no access to the ground truth. Overall, tACS can entrain neural activity at a microscopic and macroscopic level.

## 3 Secondary (offline) neurophysiological effects of tACS

In the bigger picture of potential clinical applications, tACS can only be effective as a therapeutic intervention if it induces effects that last beyond the stimulation duration. The majority of research studies currently has focused on the after-effects of a single session of tACS and have suggested that tACS can induce short-term plasticity as a secondary effect of tACS besides entrainment (during stimulation). Previous studies have shown neurophysiological after-effects of single-session tACS lasting for an hour or more across various cortical regions (Moliadze et al., 2012; Neuling et al., 2013; Strüber et al., 2015; Kasten et al., 2016; Stecher et al., 2017; Moliadze et al., 2019; Wischnewski et al., 2019a; Ghafoor et al., 2022). Kasten et al. (2016) applied 20 min of tACS at the individual alpha frequency over the visual cortex and measured changes in endogenous alpha oscillations up to 90 min post-stimulation. The results showed that the individual alpha power was increased for 90 min, although the difference with sham tACS was significant for only 70 min due to a natural increase in alpha power over time (Benwell et al., 2019).

Wischnewski et al. (2019a,b) provided causal evidence that the observed after-effects are related to NMDA receptor-mediated plasticity. They applied 15 min of beta tACS to the primary motor cortex using a high-definition montage. In one condition, participants received tACS together with a placebo medication, while in the other condition, participants received an NMDA receptor blocker. In the tACS + placebo condition, increased primary motor cortex excitability was observed, as evidenced by increased MEP measured by applying single-pulse transcranial magnetic stimulation. Further, beta power, but not power in other frequency spectra, was significantly increased. Crucially, the effects on excitability and beta power were abolished in the tACS + NMDA blocker condition. This suggests that NMDA receptor plasticity was a key underlying factor for the observed after-effects. The effects were still observed 60 min after stimulation. The study did not contain measurements after 60 min, so the effects potentially lasted for longer.

Riddle et al. (2020) combined results from three experiments that collected EEG before and after alpha tACS. They also collected DNA to determine the presence of the Val66Met polymorphism in the gene that codes for brain-derived neurotrophic factor (BDNF). Previous studies suggest that Val66Met polymorphism diminishes responsiveness to stimulation by lowering NMDA receptor-dependent synaptic plasticity. They found Met carriers showed less of an increase in alpha activity after tACS than non-Met carriers, providing evidence for the role of BDNF and synaptic plasticity in tACS-related after-effects.

### 3.1 Spike-timing-dependent plasticity

As discussed, the primary mechanism of tACS is the entrainment of neural firing during stimulation. As such, under tACS electrodes, neural firing occurs at a particular phase compared to the surrounding local field potential (LFP). Animal and computational work has demonstrated that prolonged spike-LFP coupling is crucial for inducing spike-timing-dependent plasticity (STDP) (Caporale and Dan, 2008; Anastassiou and Koch, 2015; Andrade-Talavera et al., 2023). STDP is a form of synaptic plasticity in which the timing of action potentials between pre- and postsynaptic neurons determines whether synaptic strength is increased or decreased (Caporale and Dan, 2008; Markram et al., 2012). Several studies have used feedforward neural network models to examine the relationship between spike phase-locking and STDP. These models typically comprise integrate-and-fire neurons, where pre-synaptic populations of oscillating neurons provide input to single post-synaptic neurons (Muller et al., 2011; Masquelier et al., 2009; Luz and Shamir, 2016). Muller et al. (2011) demonstrated that precise spike phase-locking could be achieved through periodic modulation of presynaptic firing rates during oscillations in conjunction with STDP. Their findings emphasized that the balance between potentiation and de-potentiation in the STDP rule critically determines the firing phase of output neurons. Masquelier et al. (2009) demonstrated that the interaction between oscillatory input and STDP creates an efficient learning mechanism by showing that neurons could effectively detect patterns in input currents even when phase-of-firing coding was present in only a small subset of afferents. Luz and Shamir (2016) investigated how phase relationships between pre- and post-synaptic spikes influence synaptic weight dynamics. Their work revealed that the temporal structure of the STDP rule can establish a preferred firing phase in post-synaptic neurons, which proves crucial for the emergence of oscillatory behavior. Consequently, synaptic plasticity facilitates the downstream transmission of oscillatory signals along neural information processing pathways. Together, these studies demonstrate the intricate relationship between neural firing and oscillations that may result in STDP. As such, modulation of neural oscillations via tACS may induce lasting changes without affecting all neural populations uniformly.

**Figure 1:**
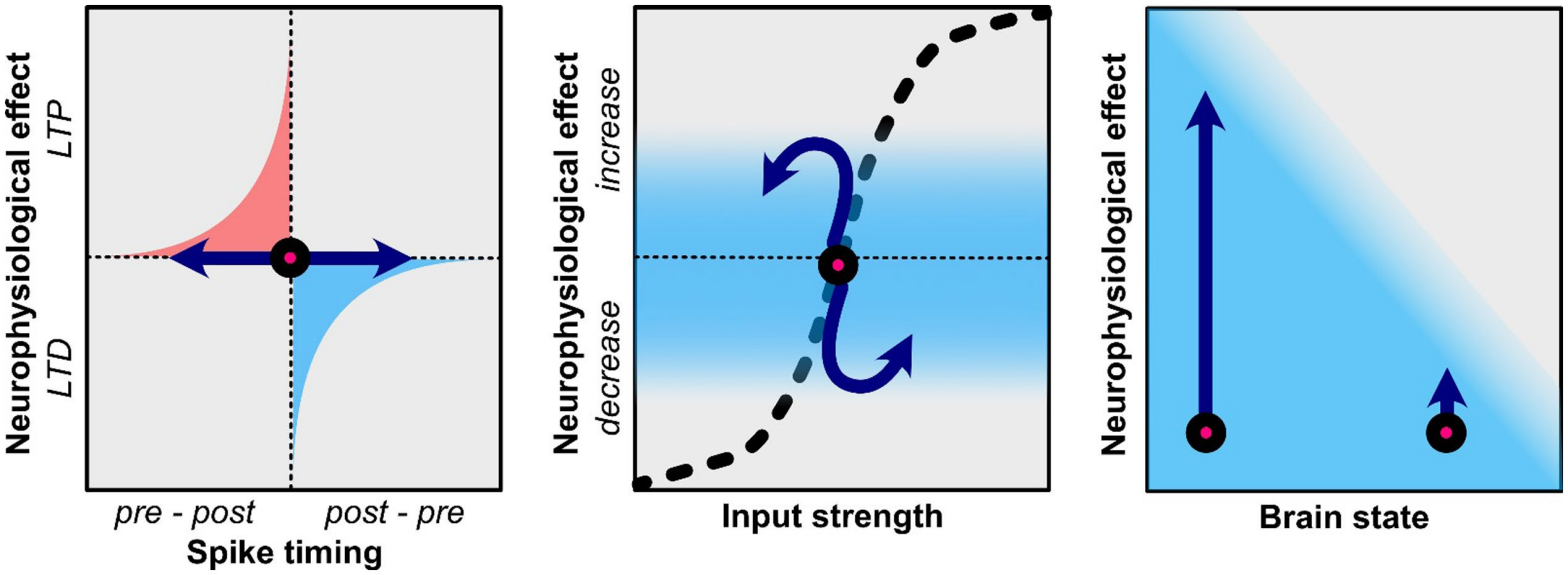
Potential neuroplasticity mechanisms related to the after-effects of tACS. Effects of tACS are shown by the blue arrows. Left: TACS applied in a frequency that is at or slightly below that of endogenous rhythms may result in long-term potentiation (LTP) as it promotes the natural sequence of pre-synaptic firing preceding post-synaptic firing. TACS that is at a higher frequency than endogenous rhythms may result in long-term depression (LTD) as it promotes post-synaptic firing that precedes pre-synaptic firing. Middle: Homeostatic properties affect the efficacy of tACS. If neural oscillations or firing synchrony is already increased tACS may not be able push this effect further and may result in homeostatic plasticity where the opposite effect is observed. Right: The current brain state also determines efficacy of tACS. While some in some physiological or behavioral state tACS may result in large changes, other psychological/behavioral states may prevent tACS from having a strong effect.

In the context of tACS, it has been proposed that STDP can be induced if the stimulation frequency is at or slightly below the endogenous oscillatory frequency (Zaehle et al., 2010; Vossen et al., 2015; Wischnewski and Schutter, 2017; Schwab et al., 2019, 2021; Vogeti et al., 2022; Pariz et al., 2023). This hypothesis rests on the observation that long-term potentiation (LTP) is promoted when presynaptic activity consistently precedes postsynaptic activity, which occurs when tACS matches the frequency of endogenous oscillations (Figure 1; Zaehle et al., 2010). In contrast, long-term depression (LTD) is related to postsynaptic activity preceding presynaptic activity, which is related to tACS at higher frequencies than the endogenous oscillation frequency (Zaehle et al., 2010). According to the tACS-STDP hypothesis, the strongest tACS after-effects would be observed at or near the individual endogenous oscillatory frequency. Several studies provide evidence in favor of this hypothesis (Kasten et al., 2016; D’Atri et al., 2017; Wischnewski and Schutter, 2017; Schwab et al., 2019; Kemmerer et al., 2022). As previously described, Kasten et al. (2016) found an increase in alpha power after individual alpha tACS. Crucially, these effects were not observed in frequencies of 3 Hz above or below the individual alpha frequency. Furthermore, Kemmerer et al. (2022) investigated the effects of tACS at the individual alpha frequency as well as two control conditions where tACS was applied ±2 Hz above and below the individual alpha frequency. Only the tACS condition that matched the endogenous oscillation rhythms resulted in a bias of alpha power toward the stimulation location. Furthermore, this shift in alpha power was related to a change in visual attention only in the individual alpha tACS condition but not in the control conditions. Schwab et al. (2019) demonstrated the frequency-specificity of tACS after-effects on interregional connectivity by applying in-phase tACS. Connectivity was increased after in-phase tACS (i.e., when the phase between two regions was synchronized) compared to anti-phase and jittered-phase tACS (i.e., when the phase between two regions was not synchronized). In a follow-up study, Schwab et al. (2021) utilized coupled neuronal network models to demonstrate how STDP explains connectivity aftereffects. It was revealed that aftereffect directionality depends on both tACS frequency and inter-regional conduction delays, with maximal effects observed during short conduction delays. These findings were subsequently validated through EEG data. Moreover, Pariz et al. (2023) conducted a comprehensive investigation of tACS influences on synaptic plasticity in heterogeneous multi-layered neuronal networks. Their model incorporated varying membrane time constants to reflect cortical neuron heterogeneity, revealing that disparities in neuronal timescales enable selective and directional control of synaptic connectivity through tACS.

Collectively, these computational studies provide valuable insights into the mechanisms underlying tACS-induced plasticity while suggesting potential strategies for optimizing stimulation protocols. The progressive refinement of these models, from simple feedforward networks to complex heterogeneous systems, has enhanced our understanding of how tACS may induce lasting physiological effects through the interaction of spike-timing and synaptic plasticity.

### 3.2 Spike-phase coupling and plasticity

As discussed above, spike phase-locking is crucially related to plasticity and may be strengthened by tACS. Besides the strength of spike-phase coupling, the specific phase may also contain information about neuroplastic processes (O’Keefe and Recce, 1993; Hafting et al., 2008; Qasim et al., 2021). O’Keefe and Recce (1993) found that hippocampal firing gradually shifts compared to the theta phase while an animal moves through a maze. As such, a shift in the preferred firing phase reflected a learning process of a particular spatial map. Various studies have linked this so-called phase precession to STDP and NMDA receptor-mediated synaptic plasticity (Thurley et al., 2008; Muller et al., 2011; Reifenstein et al., 2021; Wischnewski et al., 2024c). No direct evidence for tACS-induced spike field coupling in humans currently exists. The mechanisms seem plausible as non-human primate studies suggested that spiking locks to particular phases of the tACS oscillation (Krause et al., 2019, 2022; Johnson et al., 2020). Furthermore, indirect evidence comes from Wischnewski et al. (2024c), in which it was found that TMS-induced MEP amplitude was related to the phase of the tACS oscillation. That is, TMS-related neural firing was coupled to a particular phase. Additionally, computational modeling suggested that phase coupling was related to the modulation of NMDA weights (Zhao et al., 2024). However, more direct evidence, such as from surgical patients with invasive recordings is crucial.

### 3.3 Homeostatic plasticity

While the tACS-STDP hypothesis argues for the importance of frequency-specificity, it should also be noted that various studies have reported frequency-aspecific tACS-related after-effects (Veniero et al., 2015; Wischnewski et al., 2016; Kleinert et al., 2017; Moliadze et al., 2019; Venugopal et al., 2024). As such, it is essential to consider the dynamic nature and limited resources of the brain. Brem et al. (2014) proposed the net zero-sum model, which suggests that neuromodulatory effects are limited by energy resources. In simple terms, increasing energy expenditure in one domain may result in lower energy expenditure in other domains due to the brain’s limited resources. Enhanced oscillatory power at the tACS frequency could, therefore, result in reduced oscillatory power in other frequency bands. A related but distinct concept is the idea of homeostatic plasticity (Siebner et al., 2004; Karabanov et al., 2015; Schutter et al., 2015; Nowak et al., 2017; Agboada et al., 2020; Peterson and Voytek, 2020). While appealing, the net-zero sum model is somewhat simplified as it assumes that the brain is a linear system, which it is not. For instance, increases in the amplitude of some frequency bands are correlated with increases in other frequency bands (Jensen and Colgin, 2007; Hyafil et al., 2015). Similar to the net zero-sum model, the homeostatic plasticity hypothesis (specifically concerning brain stimulation) suggests that neuromodulatory effects cannot extend beyond the dynamic neurophysiological range (Karabanov et al., 2015; Schutter et al., 2015; Peterson and Voytek, 2020). That is, neuronal firing and synaptic inputs are regulated to prevent dysfunctionally strong or weak connections. For example, a synaptic homeostasis can be achieved by up-regulating or down-regulating synaptic strength, or by changing synaptic input (Turrigiano and Nelson, 2004; Abraham, 2008). In practice, neurostimulation aimed at enhancing a particular neurophysiological pattern may instead suppress it when operating near its upper dynamic limit, while neurostimulation aimed at inhibiting neural responses may instead enhance them when operating near the lower end of the dynamic range (Figure 1; Karabanov et al., 2015). To demonstrate homeostatic plasticity, Siebner et al. (2004) consecutively applied two neurostimulation paradigms which are thought to have inhibitory effects, namely cathodal transcranial direct current stimulation (tDCS) and 1 Hz repetitive TMS. While one might expect additive inhibitory effects, 1 Hz rTMS actually increase cortical excitability after inhibitory tDCS. As such, homeostatic plasticity prevented cortical excitability to be inhibited beyond the lower end of the dynamic range. Similarly, Agboada et al. (2020) showed no further enhancement but rather a diminution of cortical excitability when the motor cortex was stimulated with 3 mA anodal tDCS two times within a 3-h interval. Interestingly, the less intense 1 mA anodal tDCS protocol enhanced excitability with repeated stimulation, showing the lack of further enhancement with the stronger stimulation could be an upper homeostatic ceiling that regulates plasticity. For tACS this could imply that increasing an already robust neural oscillation may result in after-effects where this oscillation is decreased. Despite several tACS studies observing this phenomenon (Wischnewski et al., 2016, 2021; Pahor and Jaušovec, 2018; Alexander et al., 2019; Zarubin et al., 2020; Riddle et al., 2022), homeostatic plasticity after tACS is yet to be systematically investigated.

### 3.4 State-dependent plasticity

When investigating neuroplastic effects of tACS it is also crucial to consider the current state of the brain (Kasten and Herrmann, 2022; Schutter et al., 2023). Brain states can fluctuate due to physiological or biological processes (e.g., circadian rhythm, neural oscillation phase, etc.). Alternatively, brain states are affected by behavioral and cognitive states. Examples of these behavioral states are being at rest or in an action, eyes open or closed, states of attention, cognitive effort, and many more. Behavioral states can typically be manipulated in experimental settings, while often brain states are often not controllable. Thus, since behavioral states are used to modulate brain states, they are often difficult to disentangle.

While tACS may induce plastic effects at a particular brain or behavioral state, it may have no effect when it is in another state. For instance, an increase in alpha power and coherence in the visual cortex is observed after alpha tACS only when participants have their eyes open, while no effects are observed with closed eyes (Neuling et al., 2013; Ruhnau et al., 2016; Wang et al., 2022). As such, this example reflects an interaction between behavioral and brain states that influence the efficacy of tACS. While the previous example related to oscillation power, others have shown that oscillation frequency may also depend on the state. Feurra et al. (2013) showed that MEPs induced by TMS increase after beta tACS when a person is at rest, which is consistent with other findings (Wischnewski et al., 2019b). However, when participants performed motor imagery not beta, but theta tACS resulted in increased MEPs. Furthermore, during action observation, not beta tACS but alpha and gamma tACS increased MEPs (Feurra et al., 2019). Taking into account the current brain and behavioral state is of particular importance when using tACS in a clinical setting. Riddle et al. (2022) have shown that tACS in patients with major depressive disorder can reduce alpha power, but this effect is associated with positive stimuli only, while no alpha modulation was observed during the presentation of negative stimuli.

### 3.5 Indications of plastic effects in macroscopic modalities

Changes in EEG power, either frequency-specific or frequency-specific, may hint at neuroplastic changes (Bibbig et al., 2001; Zarnadze et al., 2016; Burgdorf et al., 2019; Gefferie et al., 2021). However, EEG reflects macroscopic changes in neural activity, while synaptic plasticity occurs at the cellular level (Buzsáki et al., 2012; van Bree et al., 2024). As such, changes in EEG power from before to after tACS do not reflect plastic changes *per se*. Vice versa, the absence of changes in EEG power from pre- to post-tACS does not necessarily suggest that no plasticity occurred. The same holds for magnetoencephalography (MEG). For instance, Aktürk et al. (2022) found increased resting state power when tACS was applied at the individual theta frequency but not when tACS was applied 1 Hz below the individual theta frequency. Conversely, behavioral performance on a visual memory task increased from before to after tACS when the stimulation frequency was 1 Hz below the individual theta frequency but not when it was exactly at the individual theta frequency. As such, different stimulation frequencies resulted in distinct electrophysiological and behavioral after-effects. TACS also has been shown to have after-effects on neuroimaging, including functional magnetic resonance imaging (Alekseichuk et al., 2016; Cabral-Calderin et al., 2016; Vosskuhl et al., 2016; Chai et al., 2018). These effects appear in the stimulated area as well as distal areas.

Together these observations have two implications. First, null-results in physiological measures (EEG, fMRI) do not necessarily imply that tACS had no effect on brain functioning. Rather, potential physiological changes may be invisible to these neurophysiological recording methods. More research and novel methods are required to distinguish true null-results from seeming null-results. Second, and related to the first, tACS may induce behavioral changes in the absence of changes in EEG and fMRI. While inconsistencies between behavioral and physiological measures is often suggested as a limitation, it is to be expected given the limited sensitivity of neurophysiological recording methods.

## 4 Long-term effects of tACS

### 4.1 Neurophysiological evidence for long-term effects

Long-term or late-phase LTP-like plasticity (L-LTP) which usually refers to LTP-like plasticity lasting for longer than a couple of hours has been previously reported for other non-invasive brain stimulation (NIBS) techniques such as tDCS in the human motor cortex (Agboada et al., 2020). Similar to techniques used in other NIBS techniques like rTMS and tDCS a single session is typically not sufficient to induce L-LTP-like effects. Rather daily application for a couple of weeks may be effective (Laste et al., 2012; Grover et al., 2021, 2022; Pantovic et al., 2023). Currently, to the best of our knowledge, there exists no systematic studies on the neurophysiological mechanisms responsible for long-term effects of tACS in humans. However, many studies that use repeated or spaced stimulation technique have shown long-term effects (Grover et al., 2021, 2022; Yang et al., 2023). For instance, Grover et al. (2022) utilized spaced repetitive stimulation protocol with HD-tACS to enhance working memory. They randomized participants into two groups that received either synchronous or asynchronous theta-gamma tACS in the parietal, and dorsolateral prefrontal cortices (DLPFC) for 20 min each day for four continuous days. Immediate after-effects of the stimulation were seen in WM performance as well as 1 month post-stimulation. The strength of these effects was however related to the baseline of participants’ performance – those with lower baseline WM performance pre-stimulation performed considerably better in the post-stimulation measurements (Grover et al., 2022). The longer effects seen in this study could be due to the accumulative effects of repetitive stimulation. However, currently little is known about the physiological effects and parameters of tACS that relate to L-LTP. Even less clear is how these mechanisms translate from healthy young participants to the diseased brain. While clinical trials have shown the therapeutic value of tACS (Elyamany et al., 2021; Riddle et al., 2022), it remains to be seen if these effects observed in the clinic are a direct translation of what has been shown in healthy population.

### 4.2 Long-term effects of tACS in clinical trials

Clinical trials are important to extend the usefulness of tACS from the bench to the bedside. Currently few of these exist, as the ‘standard’ for using tACS in the clinic is only now getting defined by findings from early studies (Elyamany et al., 2021; Frohlich and Riddle, 2021).

First, for a seamless transition from pharmacological methods to NIBS, the magnitude and duration of therapeutic effects of tACS must be at the very least equal to what is normally observed with standard pharmacological therapies. This reduces the need to justify the utility of these novel therapies beyond the arguments of less comparative side-effects. Secondly, long-term tACS effects in the clinic, like what has been observed in healthy participants, helps to better understand the basic neurophysiology of cortical regions in diseased states, which can improve treatment methods.

In this section, we summarized clinical trials using tACS with a bias for trials with longer follow-ups (after-effect measurements). While these trials with tACS are still in their infancy, we hope a review of them will shed some light on what progress has been made, which might be useful for future clinical applications of tACS. The aim therefore is to explore the long-term after-effects of tACS in clinical trials, examining the methodological choices and the measurement outcomes. While we made some references to tDCS and rTMS were necessary, a detailed comparison of the effect of these techniques in neuropsychiatric disorders is beyond the scope of this review.

#### 4.2.1 Depression

Abnormalities in theta (Jaworska et al., 2012), alpha (Thibodeau et al., 2006; Gollan et al., 2014), beta (Shen et al., 2017; Cai et al., 2018) and gamma (Nugent et al., 2019) oscillations have been reported in major depressive disorder (MDD).

In the first double-blind randomized clinical trial to examine the efficacy of tACS as a treatment for MDD symptoms, Alexander et al. (2019) applied 1 mA tACS for 40 min with a bilateral montage (F3, F4, and Cz montage) in the DLPFC for 5 consecutive days. Thirty-two patients were randomized into three groups – sham, 10 Hz, and 40 Hz stimulation. They found patients receiving the 10 Hz tACS had significant reduction in depressive symptoms compared to the sham and 40 Hz groups as measured by the Montgomery-Asberg and the Hamilton Depression Rating Scales (Alexander et al., 2019). At 2-week follow-up, the 10 Hz group showed significant reduction in MDD symptoms compared to sham and 40 Hz groups. This reduction was stable for all three groups at 4-week follow-up though no significant differences between the groups were observed. Building upon these findings, Riddle et al. (2022) investigated the modulation of individual alpha frequency in 84 MDD patients using 1 mA 10 Hz tACS in another double-blind randomized placebo-controlled clinical trial. With five consecutive days of stimulation, a decrease in the left frontal individual alpha frequency was observed for active tACS compared to the sham group. These reductions were strongest for patients with severe depression as well as those who were on antidepressants. To further understand how frontal oscillations interact with the tACS (to demonstrate the context-dependency of stimulation effects), patients were presented with positive, neutral, and negative images. Without stimulation, patients with MDD showed an elevated response in their left frontal alpha power, but a reduction for the stimulated group (Riddle et al., 2022). While no long-term follow-ups were conducted in this study, their conclusions demonstrate the potential for effective modulation of alpha oscillations when paired with medication as a better treatment for MDD compared to medication alone. This also confirms other studies that found a greater degree of response in patients with medication and stimulation compared to those with only medication (Wang et al., 2022; Zhou et al., 2024). Recently, Zhou et al. (2024) investigated tACS as an add-on treatment for patients with MDD. Patients undergoing MDD treatment with escitalopram (10–20 mg daily) were stimulated for 40 min once daily 77.5 Hz tACS for 20 days. Improvements in depressive symptoms were indexed by the Hamilton rating scale for depression (HAMD-17) scores. After 4 weeks of stimulation, the tACS group had higher mean reduction on the 17-item HAMD-17 scale compared to the sham group. This reduction in depressive symptoms (lower mean scores) was prevalent at the week 8 follow-up. The study by Zhou et al. (2024) demonstrates not only the cumulative potential of combining stimulation with medication but also the long-term plasticity-inducing effect. Furthermore, repeated stimulation of twice daily or more for many days might be a good way to induce prolonged after-effects, compared to single session protocols that do not lead to any changes in depressive symptoms (Palm et al., 2022).

#### 4.2.2 Schizophrenia

Alpha rhythms are predominantly responsible for many tasks in an awake individual, such as attention, memory, perception, consciousness, and visual processing (Buzsáki and Draguhn, 2004). The ubiquitous nature of these oscillations makes their dysfunction a major problem, often resulting in different neuropsychiatric disorders. Previous tACS studies have documented the efficacy of modulating the alpha oscillations in the brain (Zaehle et al., 2010; Neuling et al., 2013; Vossen et al., 2015; Kasten et al., 2016). tACS targeting alpha and gamma reduced the prevalence of auditory hallucinations, negative symptoms, and improved cognition (Farcas and Iftene, 2022).

In a case report, Sreeraj et al. (2019) applied 2 mA theta tACS (6 Hz) in 5 sessions for 20 min each in a patient with paranoid schizophrenia. Working memory was assessed with n-back task. Post-stimulation evaluation revealed an improvement in working memory that was stable for 50 days. In a follow-up study, 12 schizophrenia patients with persistent delusions were stimulated with 2 mA 10 Hz tACS twice daily for 5 days (10 sessions). A reduction in delusions severity, and positive and negative symptoms were found, that did last for 1 month post-intervention (Sreeraj et al., 2020).

Mellin et al. (2018) conducted one of the first double-blind randomized sham-controlled clinical trials where the neuromodulatory effects of tACS were compared to tDCS in schizophrenia patients with persistent auditory hallucinations. Twenty-two patients were randomized into three groups – 10 Hz tACS, tDCS or sham tACS. Stimulation was delivered in a frontal bilateral (F3/F4) montage twice daily for 20 min each over 5 consecutive days, while the auditory hallucination rating scale (AHRS), positive and negative syndrome scale (PANSS), and brief assessment of cognition in schizophrenia (BACS) where used to assess clinical symptoms of the disease. As there were no significant differences between the three groups post-stimulation, the authors used raw effect sizes to show the neuromodulatory changes induced by the stimulation (tACS and tDCS) and sham. Within the 5 days of stimulation, tACS had the largest effect sizes on the AHRS scale, while tDCS showed largest effect sizes on the PANSS and BACS scales. Follow-up changes in effects sizes after 1 month was likewise different for each stimulation group.

In another randomized double-blind sham-controlled trial, Ahn et al. (2019) stimulated the temporal lobe of 22 schizophrenia patients with 10 Hz tACS twice daily for 5 days. They reported an enhancement of alpha oscillations which further enhanced the 40 Hz auditory steady-state response, leading to an improvement in auditory hallucinations. Importantly, these effects were stable for one-month post-stimulation, demonstrating alpha tACS can modulate networks long-term.

#### 4.2.3 Stroke and neglect

TACS has also been shown to have beneficial and lasting effects in recovery after stroke (Naros and Gharabaghi, 2017; Schuhmann et al., 2022; Grigutsch et al., 2024; Middag-van Spanje et al., 2024). The effects of tACS and neglect training were compared with sham to assess the cumulative efficacy of tACS in a double-blind, randomized, placebo-controlled trial by Middag-van Spanje et al. (2024). In a 6-week period, 18 sessions of 40 min of 10 Hz tACS or sham tACS combined with visual scanning training were administered in 22 visuospatial neglect patients. Assessment of stimulation and training efficacy was done immediately after the first, ninth, and 18th training sessions as well as 1 week and 3 months after the completion of the trial. Compared to the sham group, patients with the combined training and tACS showed significantly improved visual search and visual detection performance in their neglected side (Middag-van Spanje et al., 2024). Grigutsch et al. (2024) tested the effects of theta-gamma peak-coupled 75 Hz tACS on motor skill acquisition in 78 young healthy participants and 20 stroke survivors. They found no significant effect of the stimulation on general motor skill acquisition in the healthy participants and surprisingly, a diminished motor skill acquisition in the stroke survivor group. This single session result lends credence to a previous study that did not also find any improvement in patients when non-repetitive/cumulative stimulation method was used (Palm et al., 2022). Overall, while beneficial effects of tACS for stroke recovery may exist, more systematic research is required.

#### 4.2.4 Parkinson’s disease

Parkinson’s disease (PD) has been another field that has sprouted a variety of tACS studies. A seminal study by Brittain et al. (2013) has shown that tACS that is in the same frequency as tremor but in an opposing phase can ‘cancel out’ tremor symptoms. They have reported a tremor reduction of up to 50%. Besides the tremor frequency, another target frequency may be the beta rhythm as studies on PD suggests the depletion in dopamine signaling creates a ‘run-away’ cortical oscillation in the beta frequency range in the basal ganglia (Brown, 2006; Little and Brown, 2014; Neumann et al., 2017). This has led to the hypothesis that externally regulating the beta frequency in the brain via tACS might counterbalance the maladaptive plasticity in PD (Krause et al., 2014; Del Felice et al., 2019; Madrid and Benninger, 2021; Guerra et al., 2022). In healthy participants, beta tACS (20 Hz) applied during a no-go visuomotor task slowed movement (Pogosyan et al., 2009). Long-term effects of beta tACS in clinical treatment of PD have been explored in recent studies (Del Felice et al., 2019). Del Felice et al. (2019) demonstrated the effects of beta and theta tACS in reducing tremors in PD patients using a randomized crossover trial. Patients were stimulated with 30 Hz tACS and tRNS for 5 days a week within 2 weeks in total. When compared with tRNS, tACS improved motor and cognitive performance as shown by reduction in symptoms in clinical assessments. These changes were present after the first session as well as the end of the trial.

#### 4.2.5 Memory impairments – Alzheimer’s disease and dementia

Cortical network activity is disrupted or greatly reduced in neuropsychiatric disorders. In Alzheimer’s disease (AD) for example, anatomical degeneration caused by neurofibrillary tangles and plaques led to major disruptions in the vital long-range neuronal synchrony (Van Hoesen and Solodkin, 1994; Palop et al., 2006). These disorganizations lead to the loss of olfactory (Vasavada et al., 2015; Son et al., 2021) and spatial (Kunz et al., 2015) information processing. Gamma power in the medial and lateral entorhinal cortices are reduced in a transgenic mice model of AD (Klein et al., 2016). Kim et al. (2022) used a light flicker at 40 Hz with treadmill exercise to modulate activity in the primary visual cortex of an AD mouse model. The authors reported cell apoptosis and a significant reduction of beta-amyloid and tau protein levels.

Evidence for the importance in AD was also revealed via optogenetic methods. Stimulating fast-spiking interneurons optogenetically in the mice hippocampus with 40 Hz reduces significantly the levels of amyloid plaques (Iaccarino et al., 2016). tACS applied in the gamma frequency range was thus been suggested as a potential therapeutic treatment for AD (Al Qasem et al., 2022). A recent study using mice models of AD explored the neuroplastic effects of tACS (Jeong et al., 2021). They investigated the effect of gamma tACS on synaptic plasticity in a mouse model of AD. Forty Hz tACS was applied in a bilateral montage in the frontal lobe for 20 min for two blocks of 5 continuous days each, a total of 10 days. Measurements of field excitatory postsynaptic potential (fEPSP) and Western blotting analyses were used as indices of neuroplasticity. The authors found gamma tACS significantly enhanced fEPSPs for more than 90 min post-stimulation compared to the control groups (Jeong et al., 2021).

Imaging studies using different biomarkers have revealed tACS effects in AD patients (Sprugnoli et al., 2021; Dhaynaut et al., 2022). In a series of case and pilot studies, Dhaynaut et al. (2022) found that 40 Hz tACS significantly reduced p-Tau burden (a marker for AD), and increased gamma power but no effect on amyloid plaque (Dhaynaut et al., 2022), while Sprugnoli et al. (2021) found increases in blood perfusion in the temporal lobe after tACS, which positively correlated with changes in memory function and gamma power. Furthermore, a single session of gamma tACS in mild cognitive impairment and AD patients led to significant improvement in episodic memory (Benussi et al., 2021, 2022), while, long-term after-effects were observed for repeated stimulation over several sessions (Kehler et al., 2020; Bréchet et al., 2021). Improvements were observed in episodic memory every 2 weeks over a 14-week span with home treatment with gamma tACS (Bréchet et al., 2021). Kehler et al. (2020) found that 40 Hz tACS in the DLPFC can improve memory function up until one-month post-stimulation.

## 5 Future outlook of tACS in clinical trials

### 5.1 Optimization via systematic titrations

Similar to other NIBS techniques, tACS parameters critically influence neuroplasticity. Dosing for tACS must be re-examined thoroughly for the purposes of optimizing and extending the induced after-effects (Wu et al., 2021). Currently, there exist no systematic or standardized parameters for all disorders (Frohlich and Riddle, 2021). In many of the clinical trials reviewed above, the parameters were chosen based on previous pilot studies in small samples. The choice of electrode montage, stimulation current amplitude, duration of stimulation, number of repeated sessions, as well as the specific task all act synergistically to produce desired after-effects. Increasing current intensity might be helpful to prolong after-effects of tACS, while some configurations of electrode montage led to a better current distribution than others (Huang et al., 2017). In many of the clinical trials, the stimulation duration of 20–40 min per session was used. There is therefore a need for systematic studies that evaluate stimulation duration in the context of clinical application to find out whether short or long stimulation per session will lead to better neuroplastic outcomes.

### 5.2 Personalization of tACS via e-field modeling and closed-loop approaches

Another important aspect of optimizing tACS for clinical application is the use of simulations and modeling of current flow in the brain (Bikson et al., 2010; Datta et al., 2016; Huang et al., 2017; Van Hoornweder et al., 2023; Baetens et al., 2024; Wischnewski et al., 2024a). This method has already been used extensively in experimental research, where the parameters are carefully chosen and current flow is modeled to ascertain the outcomes (Huang et al., 2017), but often lacking in clinical trials. For specific neuropsychiatric patient populations, knowing how current flows in the diseased brain will help to fashion out parameters for that specific disease condition and sample (Boayue et al., 2018; Suen et al., 2021; Razza et al., 2023). The heterogeneity in patient samples as well as in-group differences makes it difficult to utilize the same or similar parameters across samples/populations. For instance, neurological damage can have a major effect on electric field distributions (Manoli et al., 2017; Mantell et al., 2021; Evans et al., 2023; Yoon et al., 2024). Ultimately, individualizing treatment parameters of tACS might be the solution to the lack of response observed in some patient samples.

Furthermore, closed-loop systems are currently been explored for experimental research (Wischnewski et al., 2024b; Zrenner and Ziemann, 2024), where there is a control over parameters of tACS based on the current brain state of participants. Closed-loop tACS is currently being explored by various groups (Brittain et al., 2013; Stecher et al., 2017; Ketz et al., 2018; Zarubin et al., 2020; Schwippel et al., 2024; Sousani et al., 2024). Brittain et al. (2013) adaptively adjusted tACS phase based on incoming tremor signals of PD patients. As such, in this study, the closed-loop setup relied on peripheral physiological information. Recently, Sousani et al. (2024) proposed a closed-loop stimulation approach based on brain-related physiological signals to study tACS effects in PD. In a computational approach they showed that tACS can precisely modulate neurons in deeper cortical layers, such as in the basal ganglia. Further, closed-loop tACS may have potential in the field of neuropsychiatry. In a pilot study, Schwippel et al. (2024) applied closed-loop tACS at the individual alpha frequency in 10 MDD patients for five consecutive days. They found an 80% remission rate 2 weeks after the treatment, as well as a significant reduction in alpha power. Altogether, closed-loop approaches show great potential, and future studies should further explore this method to optimize tACS parameters for efficient neurostimulation and prolongation of treatment outcomes.

The long-term effects of tACS treatment are essential for the adoption of this technique in the clinic. From the few clinical trials published so far, none investigated after-effects for longer than 3 months post-intervention. Currently, the examples of Grover et al. (2022) and others demonstrate that there exists the potential to observe long-term effects, however, for clinical populations especially, follow-ups must be tracked for at least 6 months post-intervention. Longer follow-up periods, as well as getting a better understanding of long-lasting neurophysiological changes related to tACS, are two critical points that future studies should address.

## 6 Conclusion

TACS as a neuromodulatory tool has potential clinical applications. For this to become a reality, the effects induced by the stimulation must be long-lasting. In this review, we delved into the mechanisms by which this NIBS technique operates, elucidating plausible theories, as well as the exploring some recent clinical trials. The theories of tACS mechanisms cannot be immediately validated by the clinical trials reviewed, as these clinical studies were not concerned with mechanistic questions. Mechanistically though, tACS operates via subthreshold modulation of endogenous oscillations, which leads to entrainment of cortical rhythms within the frequency band of the external stimulation. Beyond the influence of cortical oscillations, tACS can also induce a modification of synapses that results in neuroplastic changes, often governed by the general rules of homeostatic plasticity. Finally, tACS like other NIBS techniques induces effects that are heavily influenced by the brain state. Recently, application of tACS in cognitive and clinical studies have seen a dramatic increase with many novel findings that suggest that the duration of after-effects can be long. Long-term after-effects are indeed critical for the adoption of this neuromodulation technique, especially in the clinic where therapeutic use is often tied to the efficacy of stable long-lasting treatments. From the few clinical trials reviewed, tACS has shown promising results in the induction of long-term therapeutic effects in major depressive disorders, schizophrenia, Parkinson’s, stroke and neglect, and Alzheimer’s disease. It should be noted that, though tACS effects can be explained by the various theories put forth, there is currently, to the best of our knowledge no systematic clinical study that shows therapeutic effects are a result of these mechanisms. We hope future studies not only optimize parameters of tACS, as well as personalized therapeutic use but also evaluate the efficacy of tACS mechanisms in clinical applications through neurophysiological methods.
